# Indigenous Australian grass seeds as grains: macrostructure, microstructure and histochemistry

**DOI:** 10.1093/aobpla/plad071

**Published:** 2023-11-01

**Authors:** Farkhondeh Abedi, Claudia Keitel, Ali Khoddami, Salla Marttila, Angela L Pattison, Thomas H Roberts

**Affiliations:** School of Life and Environmental Sciences, University of Sydney, Camperdown, NSW 2006, Australia; School of Life and Environmental Sciences, University of Sydney, Camden, NSW 2570, Australia; Sydney Institute of Agriculture, University of Sydney, Camperdown, NSW 2006, Australia; School of Life and Environmental Sciences, University of Sydney, Camperdown, NSW 2006, Australia; Sydney Institute of Agriculture, University of Sydney, Camperdown, NSW 2006, Australia; Department of Plant Protection Biology, Resistance Biology Unit, Swedish University of Agricultural Sciences, 23053 Alnarp, Sweden; Sydney Institute of Agriculture, University of Sydney, Camperdown, NSW 2006, Australia; School of Life and Environmental Sciences, University of Sydney, Narrabri NSW 2390, Australia; School of Life and Environmental Sciences, University of Sydney, Camperdown, NSW 2006, Australia

**Keywords:** Aleurone, *Astrebla lappacea* (Curly Mitchell Grass), cell wall, *Dactyloctenium radulans* (Button Grass), embryo, endosperm, fluorescence microscopy, histochemistry, *Microlaena stipoides* (Weeping Grass), *Panicum decompositum* (Native Millet)

## Abstract

Utilization of grains of local grasses by Australia’s First Nations people for food and connection to Country has largely been lost due to colonization. Native Australian grain production has the potential to deliver environmental, economic, nutritional and cultural benefits to First Nations people and the wider community. Revitalization of the native grain food system can only be achieved if relevant properties of the grains are elucidated. This study aimed to characterize the grain structure and histochemistry of four Australian native grasses: *Dactyloctenium radulans* (Button Grass), *Astrebla lappacea* (Curly Mitchell Grass), *Panicum decompositum* (Native Millet) and *Microlaena stipoides* (Weeping Grass). For these species, as well as wheat and sorghum, whole-grain images were obtained via stereo microscopy, starch and the embryo were visualized, and sections of fixed grains were imaged via bright-field and fluorescence microscopy. The shape, size and colour of the whole native grains varied between the species. The aleurone layer was one-cell thick in the native species, as in the domesticated grains, except for Weeping Grass, which had a two-cell-thick aleurone. In the native grains, endosperm cell walls appeared thinner than in wheat and sorghum. Starch granules in Button Grass, Curly Mitchell Grass and Native Millet were found mainly in the central region of the starchy endosperm, with very few granules in the sub-aleurone layer, whereas Weeping Grass had abundant starch in the sub-aleurone. Protein appeared most abundant in the aleurone and sub-aleurone layers of the native grains, although in Button Grass, the starchy endosperm was observed to be rich in protein, as in wheat and sorghum. As a proportion of the whole grain, the embryo was larger in the native species than in wheat. The differences found in the grain properties among the four native Australian species have important implications for the agri-food industry in a changing climate.

## Teaser

Revitalization of native grain food systems in Australia requires knowledge of the structure and composition of grain components so that the grains can be utilized effectively in commercial food processing. We studied the grain morphology of four native Australian grass species that have been used as foods by Aboriginal communities for millenia: Button Grass (*Dactyloctenium radulans* ), Curly Mitchell Grass (*Astrebla lappacea* ), Native Millet (*Panicum decompositum* ) and Weeping Grass (*Microlaena stipoides* ). We characterized important macrostructural, microstructural and histochemical properties of the grains using stereo, bright-field and fluorescence microscopy. We found differences in the size, shape and colour of the whole grains, aleurone structure, spatial distribution of starch, β-glucan/cellulose, protein and relative size and location of the embryo among the four native Australian species. Our results should stimulate further research into the connections between the structural, processing and nutritional properties of these grains.

## Introduction

More than 1100 species of native grasses endemic to Australia have been identified (Penfold and Collins 2012). Seeds of some of these grasses have played an important part in the traditional diet of First Nations people for millennia, depending on the specific region and particular Indigenous culture (Tindale 1977; Pascoe 2014). In arid and semi-arid landscapes in particular, grains from grasses (mixed with seeds from non-grass species) were an important food source; they were processed into flour and cooked (Foster *et al.* 2010; Drake *et al.* 2021). In the Alligator Rivers Region of Northern Australia, it is believed that grains and seeds were likely consumed as early as 65,000 years ago (Clarkson *et al.* 2017; Pattison *et al*. in press). Many of these Australian native grasses are deep-rooted, tolerant to high temperatures and drought and well-integrated into the natural biodiversity of Australia through millions of years of evolution (Pascoe 2014; Chivers *et al.* 2015).

Wheat, rice, maize, barley, rye and oats were brought to Australia by English settlers in 1788 (Redden *et al.* 2020). Since then, almost all grain production in Australia has been from these and other annual cereal crops, such as sorghum. The systems used for cereal production in Australia have provided abundant food, feed and export income to the nation, but they have also been associated with environmental problems including soil erosion and degradation, salinity and loss of nutrients, challenging their sustainability (Hatton and Nulsen 1999; Bell *et al.* 2010; Redden *et al.* 2020).

Average wheat yields in northwest New South Wales (NSW) have typically ranged from 3 to 4 tonnes per hectare (t/ha) in recent decades. For the same region, however, estimated native grains yields vary between 0.1 and 0.5 t/ha, depending on factors such as species, rainfall patterns and management practices (Sydney Institute of Agriculture 2020). In at least some regions of Australia, native perennial grain cropping has the potential to involve less soil disturbance and greater resilience to drought, thus expanding current cereal production zones to marginal lands not suitable for annual crops including wheat and sorghum (Glover and Reganold 2010). Larger and deeper root systems in perennial grasses absorb more water than annuals, decreasing deep drainage, nitrate losses and acidification rates. The potential of perennial grasses to provide year-round ground cover also plays an important role in decreasing soil erosion (Edwards *et al.* 2015). Native grain production provides promising opportunities for commercial food applications and the involvement of Indigenous communities, which have to date been grossly underrepresented in the Indigenous foods market (Drake *et al.* 2021).

A greater understanding of the biology of candidate Australian native grasses for commercial grain production is required to promote and develop a viable native grains industry. Grass inflorescences are composed of spikelets, which themselves contain 1–40 florets. Each spikelet consists of two small sterile bracts (the glumes), which surround the florets. Each floret comprises the lemma (the outer bract), palea (inner bract) and lodicule. The palea and lemma are considered to correspond to a prophyll and a bract, respectively, whereas the lodicule contributes to opening the florets and corresponds to the petals in eudicots (Jackson and Jacobs 1985; Schmidt and Ambrose 1998; Bommert *et al.* 2005; Jacobs *et al.* 2008; Ciaffi *et al.* 2011).

The fruit of a grass is a caryopsis, which holds a single seed considered the greater part of the mature fruit. The seed consists of the embryo, endosperm, nucellus, testa (seed coat) and fruit coat (pericarp) firmly bound to the testa (Evers *et al.* 1999). The embryo is formed from an embryonic axis, consisting of the plumule and radicle, and the scutellum, which is a shield-like structure, homologous to a cotyledon, placed between the embryonic axis and endosperm. The scutellum transfers nutrients from the endosperm to the embryo (Evers and Millar 2002). The endosperm is the major storage tissue of the caryopsis and is made up of two sub-tissues: the starchy endosperm and the aleurone. Both the starch of the endosperm and the storage proteins that enclose the starch are hydrolysed by enzymes to feed the embryo upon germination. Layers of aleurone cells surround the starchy endosperm and produce these enzymes required for this mobilization (Evers and Millar 2002; Antonini *et al.* 2018).

The identity, proportions and position of the major storage components of the grain and their distribution and interactions at finer scales play an important role in determining the nutritional and processing properties of the grain and flour (Irving and Jideani 1997). The microstructure of plant-based foods, such as the degree of integrity of cell walls, can influence the digestion of dietary macronutrients. Starch, proteins and lipids are less susceptible to enzyme attack during digestion when encapsulated inside cell walls (Bhattarai *et al.* 2018).

The economic cost of grain processing is a key determinant of whether a particular species can be profitable in contemporary markets, but the amount of time and energy consumed during grain processing is easily overlooked in gauging the commercial potential of native grains (Sydney Institute of Agriculture 2020). To better understand the opportunities and constraints associated with commercial native grain production, key physicochemical properties of the grains require investigation.

This study examined the microstructure and histochemistry of the grains of four Australian native grasses: *Dactyloctenium radulans* (Button Grass), *Astrebla lappacea* (Curly Mitchell Grass), *Panicum decompositum* (Native Millet), and *Microlaena stipoides* (Weeping Grass). These species were selected based on their yield potential in their natural range, threshing simplicity (Pattison *et al.* 2023), their importance as food sources for Gamilaroi people via workshop discussions (McKemey and White 2011), and potential for commercial food applications. The potential for Weeping Grass to become a perennial grain crop in Australia has been investigated through research begun in the early 2000s (Davies *et al.* 2005; Shapter and Chivers 2015). In the former study, grain yield and its components were measured in 46 accessions of *Microlaena stipoides*. The authors found a high degree of variability among the accessions, including a 20-fold range in grain yield and a 5-fold range in grain weight. In the latter study, the productivity of nine *M. stipoides* ecotypes was assessed for grain production when grown as a companion plant with coffee or olives.

For the four native grass species listed above, the aims of this study were to analyse grain dimensions, localize and image the embryo, endosperm and aleurone layer, and determine the distribution of starch, protein and β-glucan in the grains using bright-field and fluorescence microscopy. Another Australian native grass species that was used by First Nations people as food and that has been analysed in a similar way to the current study is Kangaroo Grass (*Themeda triandra*) (Cowley *et al.* 2023); thus, we make several comparisons to Kangaroo Grass in this study. Our results were expected to link traditional knowledge of food properties of the grains with physical features at a microscopic level, explain and contextualize nutritional information associated with these grains, and provide a foundation for future research on specific food applications of these grains.

## Materials and Methods

### Plant materials

Australian native grains (mature seeds) selected for this study were from Button Grass (*Dactyloctenium radulans*), Curly Mitchell Grass (*Astrebla lappacea*), Native Millet (*Panicum decompositum*) and Weeping Grass (*Microlaena stipoides*) (Table 1). Sorghum (*Sorghum bicolor* cv. Buster) and wheat (*Triticum aestivum*) grains were used as domesticated comparator grains. The grains from Button Grass and Native Millet were harvested by hand from around 100 plants in each case, and the seeds pooled.

**Table 1.** Scientific names, basic properties and distribution in Australia of the native grain species used in this study.

Species	Scientific name	C3/C4	Plant height (cm)	Spikelet length (mm)	Distribution in Australia
Button Grass ^1^^,^^2^^,^^3^	*Dactyloctenium radulans*	C4	Up to 20	5	All states
Curly Mitchell Grass ^1^^,^^2^	*Astrebla lappacea*	C4	Up to 100	7–13	All states except Victoria
Native Millet ^2^^,^^3^^,^^4^^,^^5^	*Panicum decompositum*	C4	Up to 100	2.5–3.5	All states
Weeping Grass ^1^^,^^2^^,^^6^	*Microlaena stipoides*	C3	10–100	10–40	Eastern and Southern Australia

^1^ Cavanagh *et al.* (2019).

^2^ Jacobs *et al.* (2008).

^3^ Rose and Rose (2012).

^4^ Edwards *et al.* (2015).

^5^ Turner (1895).

^6^ Lamp *et al.* (1990).

Button Grass and Native Millet seeds were collected from the University of Sydney Plant Breeding Institute and Llara farm, both located in Narrabri (latitude: −30°18ʹ60ʹʹ S; longitude: 149°45ʹ60ʹʹ E) in northern NSW, Australia. Curly Mitchell Grass seeds were obtained from Thallon (latitude: −28°38ʹ60ʹʹS; longitude: 148°51ʹ60ʹʹE), Queensland and Weeping Grass seeds were supplied by Creative Native Food Service Co, Australia, harvested from a farm outside of Armidale (latitude: −30°30ʹ30ʹʹ S; longitude: 151°40ʹ16ʹʹE), NSW in May 2021. Seeds were threshed with methods best suited to each species (Pattison *et al*. in press), cleaned manually by sifting and winnowing to remove impurities such as chaff and dust and maintained at 4 °C until analysis.

### Grain shape, size and weight distribution

For external morphology analysis, images of the whole grains were taken using a Leica MZ16FA fluorescence stereomicroscope (Australian Centre for Microscopy and Microanalysis, University of Sydney). Microscopy images of 20 grains of each native species were used to determine grain lengths and widths using Fiji ImageJ ver1.54c. Weights of 20 seeds of each species were measured using an analytical balance with a readability of five decimal places.

### Grain microstructure and histochemistry

Grains were fixed in a 4 % formaldehyde solution at 4 °C for 24 h, sectioned longitudinally to 2–3 mm thickness and fixed again overnight. The sections were washed in distilled water (3 × 30 min) and dehydrated with an increasing ethanol series: 30 %, 50 %, 70 % and 90 % (30 min each), then with 100 % ethanol (3 × 30 min). Samples were then infiltrated with LR White resin at 25 %, 50 %, 75 % (12 h each) and 100 % (2 × 12 h). Samples were then positioned in Beem embedding capsules, filled with 100 % LR White resin to the top and polymerized at 60 °C (48 h) (Zhao *et al.* 2016).

Longitudinal sections (1 µm) were prepared with a Leica EM UC7 ultramicrotome using a Diatome histo diamond knife and collected on microscope slides. For bright-field microscopy, sections were stained with 0.1 % methylene blue for 2 min to observe cell walls (blue) and starch (white). For fluorescence microscopy, sections were stained with 0.1 % acid fuchsin for 4 min, which stains protein red, followed by staining with 0.01 % calcofluor white for 1 min, which stains cell walls blue if they contain β-glucan or cellulose (Nicholas *et al.* 1994; Kamal-Eldin *et al.* 2009; Herburger and Holzinger 2016). After each staining, sections were washed under running tap water for 1 min and dried at room temperature. The sections were mounted with one drop of glycerol, a coverslip added, and imaged using an Olympus VS120 slide scanner at 40× magnification. The images selected for the figures in the manuscript were based on intactness of the sectioned tissue, a lack of shrinking or other damage, and consistent staining over the whole section.

### Starch distribution

Grains were cut transversely using a scalpel. The sections were suspended in 25 % Lugol’s iodine solution (30 s) at room temperature and observed under a Leica M125 C stereo microscope. Starch stained with Lugol’s iodine turned black immediately (Zhao *et al.* 2022).

### Visualization of the embryo

The embryo of each of the native grains was visualized using vital staining. Grains were cut longitudinally through the embryo with a scalpel and then immersed in 1 % (w/v) 2,3,5-triphenyltetrazolium chloride (TTC) solution and placed in the dark for 2 h at room temperature. Microscopy images were taken using a Leica M125 C stereo microscope. Viable embryos turned orange to bright red (Vujanovic *et al.* 2000).

## Results and Discussion

### Whole-grain shape, size, weight and colour

**Figure 1.**
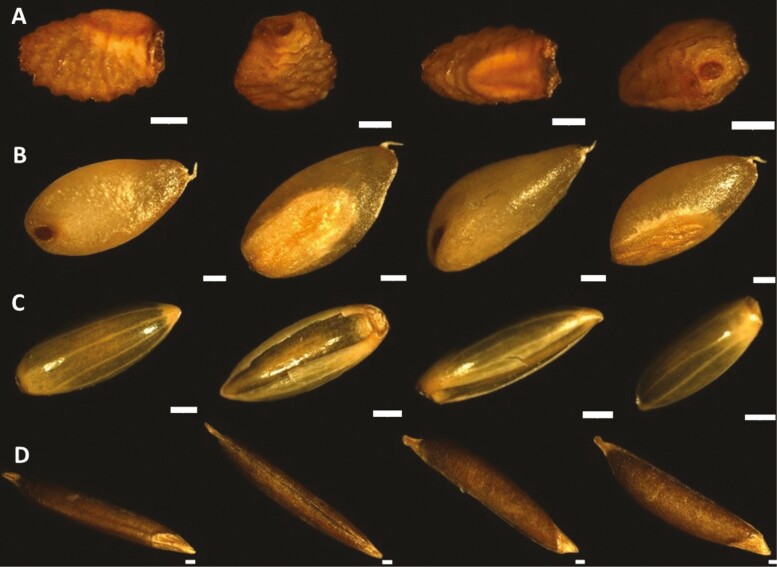
External appearance of the native grains. Images of the whole grains of (A) Button Grass, (B) Curly Mitchell Grass, (C) Native Millet and (D) Weeping Grass from different angles using a Leica MZ16FA stereomicroscope (scale bar = 100 µm). Note the shiny and stripey palea and lemma surrounding the Native Millet grain.

**Figure 2.**
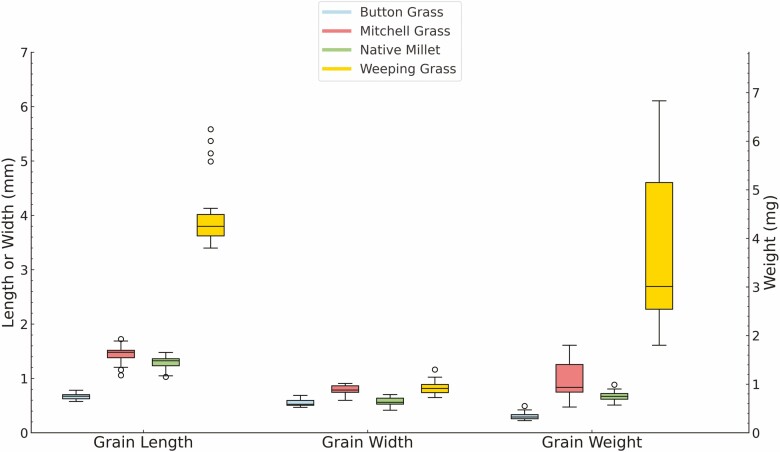
Box plot analysis of grain length, width and weight of the four native grasses (*n* = 20). Length and width (in mm) of the grains were determined using a Leica MZ16FA stereomicroscope and FIJI ImageJ software. Weight of the grains was measured using an analytical balance with a readability of five decimal places. Each box plot represents the distribution of values for Button Grass, Curly Mitchell Grass, Native Millet and Weeping Grass, respectively. The horizontal line within each box indicates the median value, while the upper and lower borders show the interquartile range (25th to 75th percentiles). Whiskers extend to the 5th and 95th percentiles. Points outside the whiskers are outliers.

The shape, colour and size of whole grains, imaged from four angles to highlight different structures, varied between the four native grain species (Figs 1 and 2; Supporting Information—Table S1). Button Grass grains had an ovoid shape, whereas Native Millet grains tended to be long and ellipsoid. Curly Mitchell Grass and Weeping Grass grains were found to be pyriform and canoe-shaped, respectively (Fig. 1). The smallest and largest (and heaviest) grains among the species were those of Button Grass and Weeping Grass, respectively, the latter being highly elongated (Fig. 2), like that of Kangaroo Grass (Cowley *et al.* 2023). The colour of the seeds varied from yellow-brown in Curly Mitchell Grass, orange-brown in Button Grass and dark brown in Weeping Grass to shiny dark brown or black in Native Millet.

The shiny appearance of the Native Millet was due to the retention of the lemma and palea, despite applying the most effective threshing method. Fig. 1C reveals how tight and far the lemma is wrapped around the palea, and thus why these structures are so difficult to remove. Historically, it was common for Indigenous people to incorporate the lemma and palea of Native Millet grain into the final flour (Latz 1995). In oat grain, this results in an increase in the fibre and a decrease in digestible carbohydrate content (Ganssmann and Vorwerck 1995). The Cooper Creek people of Central Australia used two stones—a large, uneven slab and a smaller, ball-shaped one—to grind the whole seeds of Native Millet into a meal, as reported by Gregory in 1887. The seeds were placed on the larger stone and then ground into a meal, sometimes using water to aid in the process (Tindale 1977; Clarke 2011).

Native Millet seeds can be ground, with the resulting flour absorbing water (i.e. caking) readily. However, grinding Native Millet seeds is more difficult than for seeds of other species that are both soft and readily fractured (Mildwaters and Clarkson 2020). In a study by Jenifer *et al.* (2023), Native Millet wholemeal flour had a poorer ability to form a paste with water, which then had a lower viscosity, compared to wheat cv. Spitfire wholemeal flour. This was attributed to the relatively lower starch and higher fibre content found in Native Millet seeds, which were threshed to a similar extent as in traditional methods (Jenifer *et al.* 2023).

### Endosperm starch, cell wall components and protein

**Figure 3.**
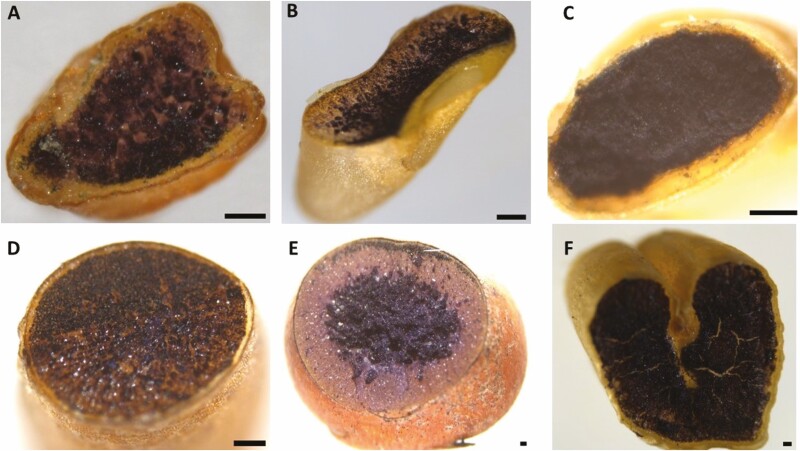
Starch distribution in the native and domesticated grains. Grains of (A) Button Grass, (B) Curly Mitchell Grass, (C) Native Millet, (D) Weeping Grass, (E) sorghum and (F) wheat were cross-sectioned using a scalpel and infused with 25 % Lugol’s iodine for 30 s at room temperature, staining the starch black. The grains were observed using a Leica M125 C stereo microscope (scale bar = 100 µm).

Most of the metabolizable energy stored in the endosperm is in the form of starch, non-starch polysaccharides and protein. Starch is stored in the form of granules, the properties of which (such as size distribution) have effects on digestibility and nutrition. Variability in functional characteristics of the starch granules, including water absorption, swelling, pasting and gelling behaviour, as well as susceptibility to enzyme attack, is affected by their natural polydispersity (Wang and Copeland 2013). In Button Grass, starch granules were distributed over most of the starchy endosperm but did not extend to the aleurone layer (Fig. 3A), whereas in Weeping Grass, starch granules appeared to be distributed out to the aleurone layer (Fig. 3D). In Native Millet (Fig. 3C), the starch granules appeared to be densely packed in the starchy endosperm, as those in wheat (Fig. 3F). In Curly Mitchell Grass (Fig. 3B), the starch granules appeared to more concentrated on one side of the grain, whereas in sorghum, the starch is concentrated in the centre of the grain (Fig. 3E).

**Figure 4.**
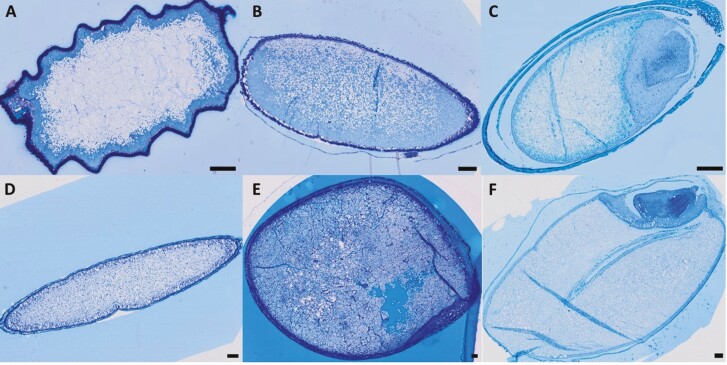
Tissue structure of the native and domesticated grains. Resin-embedded grains of (A) Button Grass, (B) Curly Mitchell Grass, (C) Native Millet, (D) Weeping Grass (E) sorghum and (F) wheat were sectioned longitudinally (1 µm) with an ultramicrotome and stained with 0.1 % methylene blue for 2 min. The tissue structure (stained) was observed using an Olympus VS120 slide scanner at 40× magnification (scale bar = 100 µm). Starch appears white, whereas cell walls (specifically beta-glucan and cellulose) appear stained. Note the palea and lemma surrounding the Native Millet seed (C).

**Figure 5.**
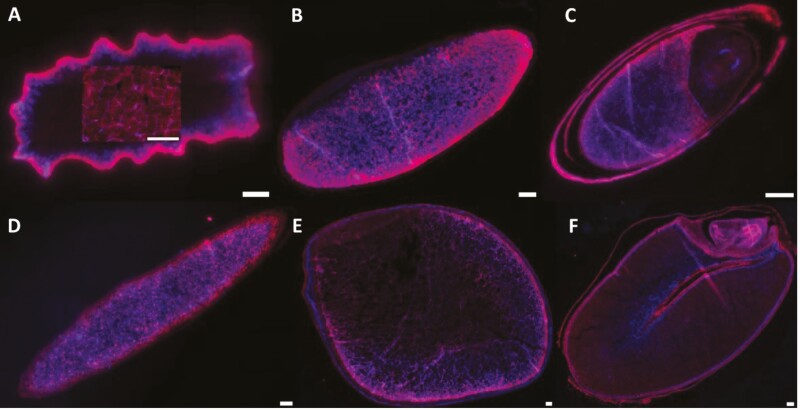
Protein and cell wall visualization of the native and domesticated grains. Resin-embedded grains of (A) Button Grass, (B) Curly Mitchell Grass, (C) Native Millet, (D) Weeping Grass, (E) sorghum and (F) wheat were sectioned longitudinally (1 µm) with an ultramicrotome and stained with 0.1 % acid fuchsin for 4 min and 0.01 % calcofluor white for 1 min. The staining showing proteins in red and cell walls (containing β-glucan or cellulose) blue, respectively, was observed using an Olympus VS120 slide scanner at 40× magnification (scale bar = 100 µm). The section in the box in the middle of the Button Grass image shows the endosperm structure at a higher exposure setting but at the same magnification. Note the palea and lemma surrounding the Native Millet seed (C). For colour figures, refer to the online version.

**Figure 6.**
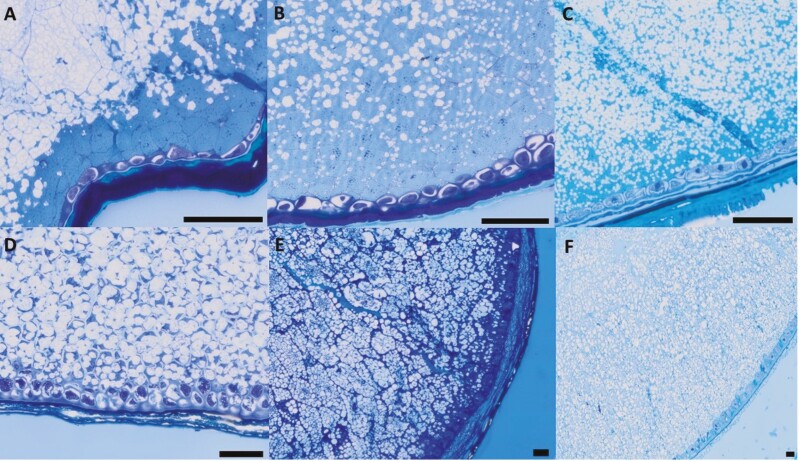
Detailed structure of the aleurone layer and starchy endosperm of the native and domesticated grains. Close-up microscopy images (based on images from Fig. 4) of longitudinal sections (1 µm) of the resin-embedded grains of (A) Button Grass, (B) Curly Mitchell Grass, (C) Native Millet, (D) Weepin Grass, (E) sorghum and (F) wheat, stained with 0.1 % methylene blue for 2 min. The staining showing the aleurone and endosperm cell structure of the grains was visualized using an Olympus VS120 slide scanner at 40× magnification (scale bar = 50 µm).

Observation of resin-embedded and sectioned grains of Button Grass, Curly Mitchell Grass and Native Millet revealed that starch granules were located in the starchy endosperm, with fewer granules in the sub-aleurone layer (Figs 4–6), as appears to be the case in Kangaroo Grass (Cowley *et al.* 2023). In contrast, starch granules in Weeping Grass were abundant in the sub-aleurone. In Button Grass, Native Millet and Weeping Grass, the size of the granules appeared to be relatively constant in starchy endosperm, as in wheat grain. In contrast, the granules in Curly Mitchell Grass were clearly smaller in the sub-aleurone layer compared to those in the central regions of the starchy endosperm, as in sorghum grain. Estimates of typical starch granule size with reference to the 50-μm scale bar are Button Grass 5 μm, Curly Mitchell Grass 5 μm, Native Millet 2–3 μm and Weeping Grass 10 μm (Fig. 6).

In all the native species, the starchy endosperm cell walls were thinner than in the domesticated grains. Weeping Grass appeared to have the thickest endosperm cell walls among the native species (Figs 4–6), which is in line with an earlier finding that weeping grass retained entire endosperm cell wall structure at maturity, as opposed to the remnants of cell walls retained in most cereals (Shapter *et al.* 2008). In Curly Mitchell Grass, Native Millet and Weeping Grass, the endosperm cell walls were strongly stained blue by calcofluor white, as in Kangaroo Grass (Cowley *et al.* 2023), β-glucan is found mainly in the endosperm of cereal grains and is categorized nutritionally as a soluble dietary fibre component, whereas cellulose is insoluble. Barley and oats are well-known to be high in β-glucan, but the amount of β-glucan present differs in both these species depending on the genotype (up to 15 % by weight for barley and up to 7 % by weight for oats). Typically, β-glucan is evenly spread throughout the endosperm of barley grains, while it is more concentrated in the outer layers of oat grain endosperm (Autio and Salmenkallio-Marttila 2001; Vasanthan and Temelli 2008).

Several nutritional studies have established a correlation between regular consumption of foods containing cereal β-glucan in appropriate concentrations and a decreased likelihood of chronic health issues. By reducing blood serum cholesterol levels, β-glucan helps mitigate the risks associated with cardiovascular disease (Braaten *et al.* 1994). Additionally, it aids in regulating blood glucose levels, thereby contributing to the management of diabetes (Wood *et al.* 1994).

Although grains of grasses generally have low cellulose contents (Fincher and Stone 2004), and thus high β-glucan in the endosperm is the more likely explanation for the observations in Fig. 5, there is an interesting exception in rice, where endosperm cell walls of mature rice grains contain significant amounts (up to 30 %) of cellulose (Shibuya and Nakane 1984).

Based on the observed red or pink staining in sections stained with acid fuchsin (Fig. 5), endosperm protein was most concentrated in the aleurone and sub-aleurone layers of all the native grains, except Button Grass, in which the starchy endosperm appeared to be rich in protein, as in wheat and sorghum grain. In Button Grass, the endosperm cell walls were stained a mixture of blue and pink, suggesting the presence of both protein and β-glucan (Fig. 5A). The red stain of the aleurone cells in all species indicated an abundance of protein in that tissue (Fig. 5).

### Aleurone layer microstructure

Cells in the aleurone layer produce enzymes required for mobilization of the endosperm during seed germination (Evers and Millar 2002; Antonini *et al.* 2018) and have significant implications for the nutritional properties and shelf life of the flour. The wheat aleurone layer is highly nutritious as it contains abundant dietary fibre as well as several classes of biologically active compounds, including phenolic antioxidants, phytate, lipids and vitamins B and E. Furthermore, the dietary fibre present in the wheat aleurone layer is classified as a non-starch polysaccharide, playing a significant role in influencing the properties of starch-based foods, such as their rheology and texture. Nevertheless, the oxidative rancidity of wheat flour containing substantial aleurone tissue poses a significant challenge during storage and production, resulting in a shortened shelf life (Jin *et al.* 2021).

The thickness of the aleurone layer (i.e. the number of adjacent cells), as well as the size and shape of the aleurone cells, varied among the native species (Fig. 6). The aleurone layer was 1–2 cells thick in Weeping Grass (Fig. 6) (Kasem *et al.* 2011), but only one cell thick in the other native species, as in Kangaroo Grass (Cowley *et al.* 2023), as well as in wheat and sorghum grain (Fig. 6). In barley, the aleurone is 2–4 cells thick (Paleg and Hyde 1964; Clutterbuck and Briggs 1973). The aleurone cells in Weeping Grass appeared to be larger compared to those of the other native species and domesticated grains (Fig. 6D). The corresponding cells in Native Millet (Fig. 6C) were more block-shaped, like those in sorghum (Fig. 6E) and wheat (Fig. 6F), compared to the other native species (Fig. 6A, B and D).

### Embryo size and position

**Figure 7.**
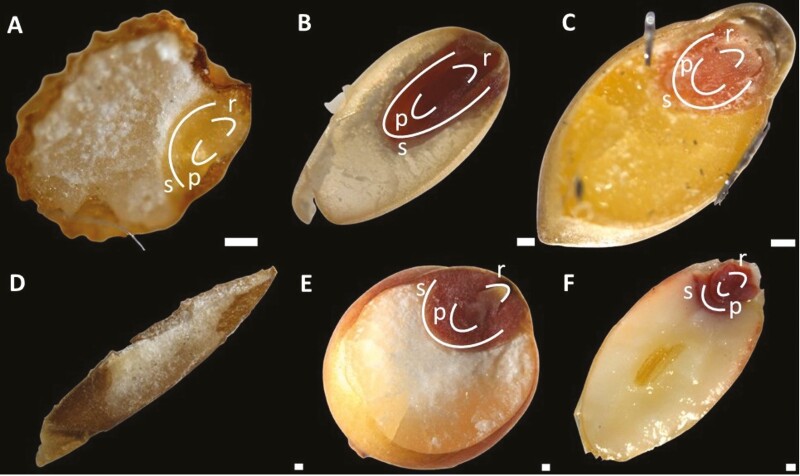
Visualization of the embryo in native and domesticated grains. Grains of (A) Button Grass, (B) Curly Mitchell Grass, (C) Native Millet, (D) Weeping Grass, (E) sorghum and (F) wheat were cut longitudinally through the embryo using a scalpel and stained with 1 % (w/v) TTC solution in the dark for 2 h at room temperature. The grains were observed with a Leica M125 C stereo microscope, showing the embryo structure, size and location. TTC stained the viable embryos orange to bright red. The images illustrate the radicle (r), plumule (p) and scutellum (s) (scale bar = 100 µm). The location of these structures in the Weeping Grass grains could not be determined.

With sections of the native grains stained with methylene blue or acid fuchsin/calcofluor white (Figs 4C and 5C), the embryo—including the plumule, radicle and scutellum—was observed only in Native Millet, which was estimated to comprise almost 40 % of the caryopsis, a larger percentage than that for wheat (Figs 4F and 5F). Based on TTC staining of longitudinally cut whole grains, the embryo of Button Grass was located closer to the edge of the grain than the embryos of the other species (including sorghum and wheat) and orientated in parallel to the edge; it was also at the top of the long edge rather than central to the shorter edge (Fig. 7A). The embryos of the other species were at the pole of the grain (Fig. 7B, C, E and F). In Weeping Grass, the embryo did not stain (~10 seeds were tested), suggesting that the seed may have been unviable (Fig. 7D), but further viability testing will be required to clarify this tentative conclusion.

Curly Mitchell Grass appeared to have the largest embryo among the native and domesticated species, constituting almost half of the grain (Fig. 7B). The health benefits of consuming foods made from grains of *Astrebla* spp. were well known to Gamilaroi, Yuwaalaraay and Yuwaaliyaay people and this knowledge was maintained within their language (Pattison *et al*. in press). The large embryo relative to the size of the whole grain of Curly Mitchell Grass (Fig. 7B) may make the flour relatively susceptible to rancidity (as embryos have a high lipid content), like in pearl millet flour, which also contains a large embryo (Nantanga *et al.* 2008).

## Conclusions

There are substantial environmental, health and cultural values among Australian First Nations people attached to the revitalization of native grain systems and building pathways for the use of native grains in modern food industries. Knowledge of the structure and composition of grain components is essential for developing industrial processes for grains. This information can complement and highlight the value of traditional knowledge when applied with respect and collaboration with traditional knowledge holders. The macrostructure, microstructure and histochemistry of the native grains reported add to previous work on nutritional composition and thus contribute to creating a basis for further research into food applications of these grains. By complementing chemical analysis, our microscopy data have aided in comprehending and visually representing structural differences in the grains of Button Grass, Curly Mitchell Grass, Native Millet and Weeping Grass, all native grasses of great importance to many First Nations peoples, including the Gamilaroi people of northern NSW. This type of analysis is applicable to other native grains in Australia (Cowley *et al.* 2023) and in other countries.

Our microscopy analysis of the four selected Australian native grain species and the localization of specific macronutrients within the caryopsis should stimulate further research into the connections between the structural, processing and nutritional properties of these grains. Specific considerations include the size and location of the embryo, the distribution of starch, β-glucan/cellulose, protein and the thickness of the aleurone layer.

## Supporting Information

The following additional information is available in the online version of this article –

**Table S1.** Grain length, width and weight for Button Grass, Curly Mitchell Grass, Native Millet, and Weeping Grass (*n* = 20; data were used to generate Fig. 2).

plad071_suppl_Supplementary_Data

## Data Availability

Raw data used to generate Fig. 2 is included as an Excel file in Supplementary Information.
